# Confederate States Hospital Reports

**Published:** 1864-04

**Authors:** 


### CONFEDERATE STATES HOSPITAL REPORTS.

### *Eleven Cases of Compound Fracture of Cranium by Gunshot Wound treated at Chimborazo Hospital.*

Case 1.—Private W. H. Hogan, company "K," 14th Virginia infantry, was wounded on the 15th of January, 1863, by the accidental discharge of a musket in the hands of a friend. The ball entering on the postero lateral-portion of the right side of the head, passed forward and upward across the parietal protuberance, emerged, making a wound about three inches in length, exposing the bone the whole length of the wound, and producing fracture of the parental bone. Was admitted on the 23d of January. At that time his mental faculties were perfect, and there was very little constitutional disturbance; no paralysis. A triangular portion of the bone had been removed, through which opening the brain was protruding. He stated that when first wounded there was complete paralysis of the left side. Cold-water dressings were applied to the wound, and a compress to the protruding portion of the brain which caused it to slough. The bowels were kept open, and the patient kept on light diet. The bone necrosed in the whole track of the ball, and was removed, exposing the brain for two inches, after which the wound healed rapidly, with a depressed cicatrix. This patient was returned to duty on the 21st of July, perfectly cured, with the exception of an occasional headache, which was generally relieved by an aperient.

Case 2.—Private H. Dixon, company "D," 30th North Carolina regiment infantry, was wounded June 30th, 1862, in the superior portion of the left parietal bone. Both tables of the bone were fractured and depressed. There was slight paralysis of the right side. He was in a dull, heavy stupor, from which it was difficult to arouse him. When aroused, he spoke very indistinctly. His mind was so much disordered he could not tell his name, nor the State from which he came. The loose pieces of bone were removed, and cold-water dressing applied. He remained in this condition for six days, at which time he began gradually to recover his mind and speech. He was furloughed on the 25th of August, almost entirely relieved of his injury and its effects.

*Remarks.*—This case is one which would seem to have demanded the use of the trephine, but, guided by the experience of Stromeyer, McLeod, and other military surgeons of high reputation, it was decided not to operate. The entire recovery of this soldier justified the omission of the operation and the course of treatment that was pursued.

Case 3.—Private H. Thompson, company "D," 16th regiment Georgia volunteers, wounded at Chancellorsville, May 3d, by buckshot penetrating left temple, just above zygomatic process, one inch posterior to outer portion of orbit. Complained of much pain in neighborhood of wound, with slight tumefaction and small discharge of healthy pus therefrom. Pulse natural. No fever. Bowels moved once daily. Cold applications were regularly kept up until May 15th, when symptoms of oppression appeared. Stertorous breathing. Respiration slow. Pulse 70. Mind much confused. Mouth and tongue quite dry. No appreciable dilatation of pupils. Orifice of wound enlarged to permit free exit to the discharge. Head shaved and ice freely applied to scalp. Purged freely; after which, one grain of mild chloride of mercury was administered every two hours.

On the 18th, patient slightly better; tongue moist; breathing natural; pulse natural; patient decidedly better Discontinued calomel, but kept cold applications to the head. From this date, patient continued to improve and apparently recovered from effects of wound, with very little evidence of impairment of intellect, until May 30th, when he suddenly became comatose, and died at nine o'clock, P. M., next morning.

*Autopsy*, on the same day, revealed a buckshot, much flattened, imbedded a quarter of an inch in base of anterior portion of middle lobe of left hemisphere of the brain. The ball had passed through the wings of the sphenoid bone, and a considerable quantity of healthy-looking pus flowed freely into the cavity of the cranium, from the temporal fossa, through the orifice. A complete sac had invested the ball and also a small fragment of bone, which was carried with it. There was very slight congestion of the "pia mater" and "arachnoid." The immediate cause of death and sudden seizure on the 30th of May were rather obscure. The most probable supposition is that pus, forming along the track of the ball, had flowed in upon the brain, through the orifice made by the ball in its entrance, instead of making its escape through the external wound, which was partially closed.

Case 4.—Private O. Ayres, company "A," 22d regiment Virginia volunteers; aged 24; occupation a farmer; wounded November 27th, 1863. Admitted, November 30th, with V. S. of right side of head, fracturing outer table of right parietal bone. When admitted, the following symptoms presented, viz: slow pulse, slight fever with coma, leading us to suspect fracture of both tables of parietal bone and pressure upon the brain; but, upon a closer examination, it was discovered that the outer table alone was fractured to the extent of one and a half inches.

*Treatment.*—Bowels were well opened with calomel and gamboge, followed by a large dose of castor oil.

November 30th.—Bowels having been well moved, patient was better. Coma, to some extent, abated. Cold applications were constantly applied to the wound, and hydr. sub. mur. pushed to ptyalism. Patient improved under this treatment, and is perfectly rational. Coma subsided.

December 5th.—Erysipelas developed itself over head and face. The parts were well painted with iodine, and tinct. ferri mur. administered internally.

9th.—Patient much improved; erysipelas disappeared; wound granulating healthy, and mind unclouded.

15th.—Patient almost entirely recovered.

25th.—Furloughed and well.

Case 5.—G. W. Gifford, company "F," 1st Tennessee regiment, wounded at the battle of Seven Pines, May 3d, 1862, by ball in right side of head, involving the coronal suture, with considerable loss of both tables of parietal and frontal bones. The brain was seen to pulsate long after the reception of injury, without the operation of trephining, and smaller pieces were thrown off at subsequent periods; but, when discharged from hospital, osseous structure had covered nearly two-thirds of original opening. Patient was returned to duty 2d February, 1864.

Case 6.—William Campbell, company "B," 42d Virginia regiment; gun-shot wound of head; ball, entering left temporal bone, was removed from left occipital bone, injuring and exposing the brain for nearly two inches.

*Treatment.*—Constant application of cold water to the head, keeping the system under the influence of morphine, and using febrifuges and cathartics as occasion required. The patient's health improved and the wound healed rapidly When furloughed, the wound was entirely covered by granulations and cicatrization, no unfavorable symptoms having intervened.

Admitted Nov. 30th, 1863. Furloughed Dec. 30th, 1863.

Case 7.—Private A. G. Powell, company "H," 4th North Carolina, wounded at Chancellorsville 3d May, 1863; gun-shot wound of head; the ball entering a little to the right of the sagittal suture, fracturing the outer table of the parietal bone. The ball, in its course, was divided by the broken edge of the bone; a part, passing in, tore away the outer plate and rested on the inner table, fracturing it and compressing the brain, causing great pain and convulsive movements. Free incision was made and the ball removed with a pair of small forceps, giving instant relief to the patient. He steadily improved and was furloughed 6th June, 1863.

Case 8.—J. Q. was wounded May 3d, 1863, by a musket ball, striking the anterior portion of the parietal bone, a little to the left of the sagittal suture. When admitted in Chimborazo Hospital, No. 4, May 6th, he was able to walk about. Some tumefaction of the scalp was apparent, and, on introduction of probe, some depression of the outer table was detected. No symptoms of cerebral disturbance. The case was treated as a flesh wound.

May 10th.—Symptoms of compression of brain: stertorous breathing, slow pulse and stupor, from which he was aroused with difficulty. Free incisions made in scalp, exposing the cranium at the seat of injury, which was found to be fractured, comminuted and depressed. Eight pieces of bone removed by elevator and forceps, without use of trephine. Some of these pieces had been forced, through the meninges, into the brain. On careful examination, the fact that no pieces of bone and no foreign substance remained in the wound was established; the inner edges of the fracture were smooth, without spicula. Edges of scalp approximated; cold-water dressings applied, and purgative doses of calomel administered, with consequent catharsis.

May 11th.—Symptoms of compression of brain have disappeared.

12th.—Suppuration freely established.

14th.—Granulation commencing; case progressing favorably.

15th.—Paralysis commencing in right arm and soon advancing to complete hemiplegia.

19th.—patient died without convulsion. Death sudden, with symptoms of spasm of larynx.

Case 9.—W. B. was wounded October 12th, 1863, by a musket ball striking the left parietal bone, about midway between top of ear and vertex, glancing a little downwards and backwards, and making its exit in a track of one and a half inches in length. Admitted in Chimborazo Hospital, No. 4, October 19th. Could give no account of himself; seemed timid, shy and easily agitated; slow of apprehension; slept but little; secretions regular; pulse 60 to 70.

October 22d.—Scalp freely incised from wound of entrance to that of exit. Cranium found to be fractured, comminuted and depressed. Portions of bone removed with probe and forceps, without trephine.

24th.—Hernia cerebri of size of common marble: the divided edges of the meninges can be distinguished upon the base of the cerebral protuberance.

26th.—The protruded brain is disappearing by suppuration.

31st.—Cerebral tumor removed by suppuration. Patient somewhat more intelligent; otherwise no change.

November 3d.—Abscess pointing over occipital bone a little to left of median line and about four inches from vertex, which was opened and discharged. On examination, fracture of occipital bone discovered; outer plate of bone elevated and a piece of lead closely impacted between the plates; all attempts to remove it without trephining ineffectual. The ball had been split when impinging upon the parietal bone, and a portion of it has passed within the cranium, making its partial exit through the occiput. The patient is improving in intelligence and in general health.

January 31st, 1864.—Patient doing well. All the wounds in scalp cicatrized; depression in parietal bone marking site of fracture; projection of outer table of occiput in place of fracture; the lead remains impacted between the tables of the bone.

Patient has recovered strength; general health good; intellectual faculties improved; he is still somewhat child-like, and is easily confused in mind.

Case 10.—Wounded June 29th, 1862. Admitted June 30th. The ball entered near the centre of the os frontis, and escaped, a week after his admission, at the posterior part of the fauces. This patient recovered promptly without any unpleasant circumstances attending his case, except the loss of the sense of smell. Furloughed July 30th, 1862.

Case 11.—Wounded December 13th, 1862. Admitted December 16th. Gun-shot wound of head, with fracture of parietal bone. When admitted, there was fungus of the brain, with paralysis of the left extremities and sphincters. With the exception of the removal of a few small pieces of bone, there was no other surgical interference in this case. He was sufficiently recovered to leave the hospital on furlough for his home, in South Carolina, on the 11th of March, 1863.

